# The Urgency of Early Diagnosis of Cancer of the Stomach1Read at the fortieth annual meeting of the Medical Association of Central New York, held at Rochester, October 15, 1907.

**Published:** 1908-04

**Authors:** Alexander McPhedran

**Affiliations:** Toronto, Ont. Professor of Medicine, University of Toronto


### The Urgency of Early Diagnosis of Cancer of the Stomach[1]

BY ALEXANDER McPHEDRAN, M. D., Toronto, Ont.

Professor of Medicine, University of Toronto

OUR views regarding the incidence and cure of internal cancer have undergone a complete change in the last decade or two, chiefly on account of the great advance in internal surgery. This advance imposes new obligations on internal medicine. The knowledge that cancer is at first a definite local disease, absolutely curable if removable, throws upon physicians the momentous responsibility of an early recognition of the disease as the first step essential to its cure.

A diagnosis to be early must be made while the disease is still localised and its complete removal therefore still possible. This point in the history of the case is usually passed long before a tumor becomes apparent, but in a few cases the infection is still circumscribed after even a large tumor has been formed. So strongly do Czerny and Rindfleisch and, later, Kraske (Boas; Diseases of the Stomach. English-American Edition; page 584) regard the outlook as unfavorable after a palpable tumor forms, that they hardly deem operation justifiable after its appearance. There are no pathognomonic symptoms of cancer of the stomach, hence we have to depend on the consideration of all the symptoms and signs.

In many cases the onset is gradual, but more often, rather sudden and attributed to errors of diet followed by acute and, later, chronic dyspepsia. Care and treatment are followed by at most only temporary relief. In a person over 40 years of age who has been previously healthy, such symptoms should arouse suspicion. In younger persons it may begin suddenly with hematemesis followed by intractable vomiting and rapid wasting.

Next to the sudden onset of digestive disturbance is the progressive decline in appetite; in time there is complete loss of

1. Read at the fortieth annual meeting of the Medical Association of Central New York, held at Rochester, October 15, 1907.

appetite and, later, repugnance to food, especially to meat. This is an important and often an early symptom of gastric cancer. The appetite is usually good, not rarely craving in ulcer, and it must not be overlooked that it is occasionally so also in cancer. Pain is not usually an early symptom, but there is often a feeling of pressure and discomfort in the epigastrium, chiefly after meals. Later pain gradually develops, but varies chiefly with the degree of gastric stasis. It is not characteristic and therefore not of much differential value.

Eructation is frequent; it may be acid, and often gives temporary relief. The gas is at first odorless, but later may become very offensive, owing to decomposing particles from the tumor. Vomiting is a frequent, but by no means a constant symptom. It is most constant as well as most copious, of course, in disease of the pylorus: it may be absent throughout, when only either curvature is affected. In acute cases vomiting may be among the first, and most distressing symptoms. In these cases, however, the progress of the lesion is so rapid that serious advance has probably been made before the severe symptoms set in. Such cases are frequent among those occurring in early life that is, before 30 years of age. Kuttner (*Berlin Klinic Wochschr.* x 44; 840) believes cancer occurs quite frequently in earlier life, and that it is often incorrectly diagnosticated.

Loss of energy, strength, and weight are usually early symptoms, occurring even before pain and vomiting. They are very suggestive if they are progressive, even under careful treatment. Anemia is usually associated with them and adds to their significance; it is probably often due to occult bleeding.

THE OBJECTIVE SYMPTOMS.

1. *Physical Examination*—In making an examination, due care should be taken to have the patient facing a good light, lying evenly on the back, with the chest and abdomen well exposed. Failure in discovering a tumor, even by good physicians, is not rarely due to want of observance of these obvious precautions. At this early stage there are rarely signs of a tumor, as one has not yet formed, but there may be signs of spasm of the walls of the stomach, as described by Cruveilhier over fifty years ago, and to which Boas has drawn attention anew, under the designation of "gastric rigidity." When present it is at first so slight as only to be felt by the hand, placed below the left costal margin, as a faint contraction of the fundus, and lasting only a moment. Later, as the contractions become more marked and rigid, they cannot only be felt, but the abdominal wall if thin can be seen to rise as a slight mound, which after a brief period disappears. When so marked as this, it gives the patient a sensation of spasm and may cause an audible gurgling sound as it disappears. For obvious reasons rigidity does not occur when the stomach is empty and is most marked if it be moderately full. It recurs at variable intervals and may usually be excited by friction especially by the cold hand. Gastric rigidity is a sign of much diagnostic value, as it indicates forcible contraction of the fundus excited by obstruction at the pylorus. It may begin fairly early, after the obstruction has developed, probably while the disease is still localised in most cases, and in time to permit of successful resection of the pylorus infection of the lymphatic glands not having yet occurred.

The stomach should be inflated in order to determine its size, position, and relation to other organs, and to a tumor if such exists. The colon should also be inflated, especially if there is a tumor of uncertain attachments. Inflation may enable us to determine the seat of the tumor, those in the pylorus before adhesion forms being movable, while in the lesser curvature they are relatively fixed. The supraclavicular glands are found enlarged in some cases, but rarely in the early stage.

2. *Examination of Gastric Contents.*—One of the most important steps is the examination of the contents of the stomach, under varying conditions, to determine the promptness of discharge of the food, the powers and rapidity of digestion, the degree of HCL secretion, the quantity of mucus present, and whether lactic acid, blood (evident or occult), pus, and tumor particles are to be found.

Free hydrochloric acid is nearly always absent in gastric cancer, even at an early stage, but it is absent also in too many people under varying circumstances to render its absence in suspected cancer more than suggestive. It is often absent in various nervous disturbances, as hysteria and neurasthenia; also in some cases of chronic gastritis and in atrophy of the gastric membrane.

In cases in which free hydrochloric acid persists, the cancer is usually regarded as forming on the base of a peptic ulcer. But excess of hydrochloric acid can scarcely confer immunity from cancer, although the acid disappears in most cases soon after the cancer begins. Three cases were cited in which free hydrochloric acid persisted almost until death, and in none of them were there any signs significant of ulceration. In one in which gastroenterostomy was done eighteen months before death the stomach wall was quite healthy. A few years ago absence of hydrochloric acid was regarded as almost a pathognomonic sign of cancer, now many regard it as of no value.

Like extreme views generally, both of these are wrong. Absence of HCL is a valuable sign in association with others, but it is only one of many we have to consider in determining the condition in any given case. The constant presence of lactic acid is strongly indicative of the existence of gastric cancer, but it does not occur until a late stage of the disease. It may be found in other diseases than cancer, but rarely if ever constantly or in copious quantities. It is found in from 75 to 90 per cent. of cases, so that "its presence speaks strongly for the existence, but its absence has no weight in favor of the absence of cancer" (Osler and McCrae). It does not appear in free quantities until hydrochloric acid disappears, and by this time there is more or less stasis of food in the stomach, and probably some degree of ulceration has taken place.

In the case of a man aged 53, with much mucus in the gastric secretion and with epigastric distress, lactic acid was present even in the fasting stomach in the morning. The protracted history of dyspepsia and the existence of chronic catarrhal inflammation rendered a diagnosis of cancer improbable. The distress was thought to be due to perigastric adhesions, and exploratory laparatomy was done in order to break them up, and if necessary to perform gastroenterostomy. Two bands of adhesions were found near the plyorus; the removal of these was deemed sufficient. He was, when dismissed, not much improved but the lactic acid had disappeared from the gastric contents.

3. *Microscopical examination*.—This is of great importance, When lactic acid is constantly present, the long bacillus first described by Boas and later and more fully by Oppler, is usually found. The precipitate of the stomach contents is best examined by being spread between two plates of glass; if held against a dark background, the solid particles can be readily seen and removed for examination. The eye of the stomach tube should also be searched for any adherent particles, as well as the material got from the tube. If nothing has been obtained from the stomach it is important, in withdrawing the tube, to compress it with the fingers, so that any contents or particles lodged in it may be obtained for examination. If ulceration has begun, pus, blood corpuscles, and bacteria, as well as small particles of tissue from the base or sides of the ulcer may be found; the latter may be so characteristic as to render an exact diagnosis possible. The odor of a particle in the eye of the tube, if fetid, may give a valuable clue.

The persistent presence of blood is a sign of ulceration. It points strongly to cancer, if associated with retention of gastric contents and defective gastric secretion. The blood should be sought for microscopically and chemically, in both stomach contents and feces. Its constant absence is strong evidence against the existence of cancer.

The motor function of the stomach deserves careful consideration. It may remain nearly normal to the end. The testing should be under identical conditions. The best course is to give an abundant meal in the evening and make an examination in the morning, a tube being passed to evacuate the stomach contents, after which the stomach should be washed out to make certain that it is quite empty.

The presence of even minute quantities of blood with pus in the contents of the fasting stomach, or of pus alone, or mixed with mucus, especially if present on repeated examinations, renders the existence of cancer almost certain. Salomon's albumin test is regarded as reliable by those who have used it. Kuttner from an extensive experience confirms its value. It is carried out by giving the patient a fluid meal, free from albuminoids, at noon, and thoroughly washing out the stomach in the evening. Next morning the fasting stomach is rinsed out several times with 12 ounces (400 cc's) of physiological salt solution, the solution being poured into, and syphoned out of, the stomach several times. The solution is then tested for albumin with Esbach's fluid. If it becomes opalescent from a flaky precipitate, the presence of albumin is demonstrated, and the existence of cancer practically confirmed. The presence of albumin is possible in simple gastritis, but Kuttner in his large experience has not met with it. That the sign is an early one is doubtful, and it is therefore not probable that it will aid in the early diagnosis.

One other method remains to be discussed, that is, the exposure of the stomach by a small incision—exploratory laparatomy. By this means not only can the condition be definitely determined in most cases, but the removal of the diseased part proceeded with, where such is advisable. To operate simply for diagnosis is, to my mind, never justifiable; to do so without having thoroughly exhausted every other means is to be unreservedly condemned. Operation should not be undertaken unless the signs distinctly indicate the organ that is affected. No "roving commission inside the abdomen" is ever justifiable, nor is operation for diagnosis in any case of advanced carcinoma, except for the relief of such conditions as gastric stasis. The aid of the surgeon should not be suggested even to our own minds until we have exhausted every means that can aid in making a diagnosis. The examinations should be full and complete, omitting no investigation that can aid in determining the condition, and they should be done promptly, without any unnecessary delay. In cases of doubt the examinations should be repeated, in some cases several times, with our minds as unbiased as if the case were a new one, as we are often led astray by the impressions already formed.

A great obstacle to laparatomy for treatment and diagnosis is the objection of the patient to it; but there is another, and that is, the hesitancy, if not the repugnance, of the physician himself. The patient's objection is often but the reflex of that of the physician. The best means to overcome both lies in the making of prompt, full, and repeated examinations, so that a definite conclusion as to the necessity to act, may be arrived at without undue delay. If such a course were generally pursued we, as well as the public, would gradually become educated in the importance of such a rational course, with the result that an increasing number of cases would be permanently relieved.

The most important step toward an early diagnosis is the arousing of our suspicions; once they are aroused, to delay full investigation when possible, would be criminal. To carry out these principles, it will be necessary to operate in all cases of gastric ulcer resisting treatment, especially after middle-life, and in those showing signs of pyloric stenosis. Even in early life it is the best course in obstinate cases. In the hands of those specially trained in gastric surgery, the results in recent years show wonderfully great success and low mortality. But operation should be advised only after the most thorough examination, and this requires fully as expert knowledge as does the operation.

In the stomach troubles of patients over 40 years of age, especially beginning after that age, we should not rest content until the diagnosis is reasonably certain that cancer does or does not exist. If the profession were fully alive to the great importance and urgency of the matter, early diagnosis would become more and more frequent. As there are no pathognomonic signs of cancer of the stomach, it is rarely possible to make more than a probable diagnosis and for this, full and repeated examinations are usually necessary. An early diagnosis is urgent as neighboring glands are liable to become infected.

151 Bloor Street.

